# On the value of preprints: An early career researcher perspective

**DOI:** 10.1371/journal.pbio.3000151

**Published:** 2019-02-21

**Authors:** Sarvenaz Sarabipour, Humberto J. Debat, Edward Emmott, Steven J. Burgess, Benjamin Schwessinger, Zach Hensel

**Affiliations:** 1 Institute for Computational Medicine, Department of Biomedical Engineering, Johns Hopkins University, Baltimore, Maryland, United States of America; 2 Center of Agronomic Research, National Institute of Agricultural Technology (IPAVE-CIAP-INTA), Córdoba, Argentina; 3 Department of Bioengineering, Northeastern University, Boston, Massachusetts, United States of America; 4 Carl R. Woese Institute for Genomic Biology, University of Illinois at Urbana-Champaign, Urbana, Illinois, United States of America; 5 Research School of Biology, The Australian National University, Acton, Australian Capital Territory, Australia; 6 Instituto de Tecnologia Química e Biológica António Xavier, Universidade Nova de Lisboa, Oeiras, Portugal

## Abstract

Peer-reviewed journal publication is the main means for academic researchers in the life sciences to create a permanent public record of their work. These publications are also the de facto currency for career progress, with a strong link between journal brand recognition and perceived value. The current peer-review process can lead to long delays between submission and publication, with cycles of rejection, revision, and resubmission causing redundant peer review. This situation creates unique challenges for early career researchers (ECRs), who rely heavily on timely publication of their work to gain recognition for their efforts. Today, ECRs face a changing academic landscape, including the increased interdisciplinarity of life sciences research, expansion of the researcher population, and consequent shifts in employer and funding demands. The publication of preprints, publicly available scientific manuscripts posted on dedicated preprint servers prior to journal-managed peer review, can play a key role in addressing these ECR challenges. Preprinting benefits include rapid dissemination of academic work, open access, establishing priority or concurrence, receiving feedback, and facilitating collaborations. Although there is a growing appreciation for and adoption of preprints, a minority of all articles in life sciences and medicine are preprinted. The current low rate of preprint submissions in life sciences and ECR concerns regarding preprinting need to be addressed. We provide a perspective from an interdisciplinary group of ECRs on the value of preprints and advocate their wide adoption to advance knowledge and facilitate career development.

## Abstract 2

This Perspective article from an interdisciplinary group of early career researchers explores the value of preprints, advocates the wider adoption of preprints to advance knowledge and facilitate career development, and tackles concerns and opportunities regarding their use.

## Introduction

The outputs of scientific research are varied, in the form of research articles, reviews, commentaries, perspectives, theory manuscripts, methods, data, reagents, model organisms, computational models, patents, drugs, vaccines, software, and highly trained researchers. Researchers are primarily evaluated on their record of peer-reviewed publications in traditional journals and the perceived value of the journal in which the work is published. This process has well-documented limitations [1–5], which provide acute challenges for early career researchers (ECRs)—graduate trainees, postdoctoral researchers, and junior group leaders, who rely heavily on timely dissemination of their work to gain feedback and recognition for their efforts.

Preprints are one mechanism to address some of these limitations. Preprints are online, freely available (open-access) scientific manuscripts posted by authors on dedicated servers prior to peer review and publication in an academic journal [66–68]. Most preprints in the life sciences are deposited concurrently with submission to a journal, yet some authors may choose preprint deposition as the sole way of communicating their work. These manuscripts are screened to contain appropriate content for the respective preprint server. Preprint servers make work immediately available to researchers because they do not perform peer review prior to dissemination.

Two of the largest preprint servers are arXiv (comprised of scientific papers in the fields of mathematics, physics, astronomy, electrical engineering, computer science, quantitative biology, statistics, and quantitative finance) and bioRxiv (repository for the life sciences). There are now over 1.3 million preprints on arXiv and approximately 40,000 preprints on bioRxiv, the latter representing the work of over 160,000 researchers from more than 100 countries. In addition, approximately 67% of bioRxiv articles posted before 2017 were subsequently published in 1,531 journals [76,80].

Facing an evolving landscape for publication and evaluation of research outputs, ECRs in the life sciences must decide how to use preprints for their work. Preprint servers in the life sciences have different scopes in terms of content, subject area, language, and geographic origin of the deposited work—multiple subjects (PeerJ preprints and bioRxiv), specific subjects (e.g., AgriXiv, PaleorXiv, PsyArXiv, ChemRxiv, EarthArXiv, EngrXiv, SportRxiv), and continent or language specific (e.g., AfricArxiv, IndiArxiv, Arabixiv, INArxiv). MedRxiv will soon focus on medicine and health sciences [36], which has shown the slowest uptake of preprints in the life sciences, with some leading medical journals not accepting submissions of preprinted manuscripts.

**Fig 1:**
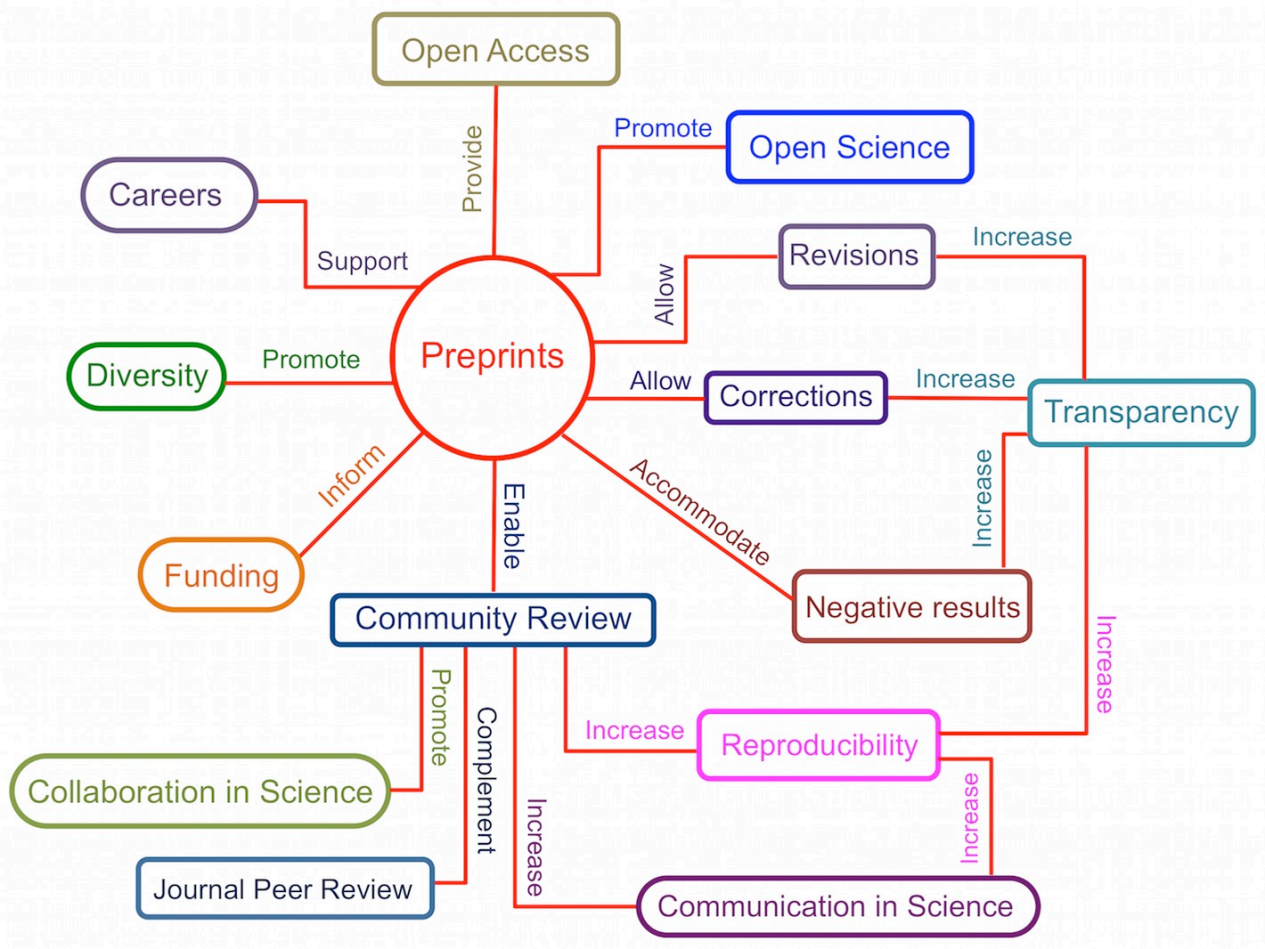
Preprints influence many facets of the scholarly landscape. Preprints are an asset for ECRs. Preprints support a vibrant research culture and impact research decisions in multiple areas of the academic endeavor. The value of preprints for the biomedical workforce and biomedical research enterprise is currently underutilized. ECR, early career researcher.

The adoption of preprinting as an academic practice has grown exponentially in recent years, and today approximately 1% to 2% of articles listed in PubMed were initially submitted as preprints [6,7,9,48]. The increasing number of biosciences preprints [7,8,9] reflects a realization that preprints can ameliorate systemic issues in journal-based peer review that disproportionately impact ECRs. We, as a group of ECRs in life sciences (Box 1), discuss here the many ways in which ECRs benefit from depositing their manuscripts on preprint servers, accelerating science communication and career progression (Fig 1).

**Box 1. Author biographies.**

Sarvenaz Sarabipour is a postdoctoral fellow in the Mac Gabhann lab at the Institute for Computational Medicine and Department of Biomedical Engineering, Johns Hopkins University. She earned her B.Sc. in Physics and Mathematics from University of Sydney, Australia; her M.Sc. from Université de Sherbrooke, Canada; and her PhD in Engineering from Johns Hopkins University. Sarvenaz builds multiscale computational models of receptor signaling networks in cell and tissue contexts. These models will enable design of specific systems-level molecular vascular interventions to control angiogenesis in diabetes and cardiovascular disease. She is an ambassador for eLife and an advocate for early career researchers, open-science, mentorship, diversity, and reproducibility initiatives.

Humberto Debat is a research associate at the Institute of Plant Pathology in the Center of Agronomic Research of the National Institute of Agricultural Technology in Argentina. Humberto studies the interface of viruses and crops from a holobiont perspective. Humberto obtained his undergraduate and graduate training in biology at the National University of Cordoba, Argentina. He is interested in novel approaches to reduce losses associated to plant diseases and passionate about understanding an expanding global virosphere. Humberto is an ambassador for eLife and ASAPbio advocating the use of preprints in life sciences.

Steven Burgess is a Carl R. Woes Institute for Genome Biology postdoctoral fellow at the University of Illinois at Urban-Champaign. Steven earned his bachelor’s degree from the University of Edinburgh. He went on to receive his doctorate from Imperial College London and did his postdoctoral work at the University of Cambridge. His research interests include photosynthesis and synthetic biology. His current focus is on optimizing the way that plants capture sunlight and use the energy for growth as part of the Realizing Increased Photosynthetic Efficiency project funded by the Bill and Melinda Gates Foundation, the Foundation for Food and Agriculture Research, and UK Aid Direct. He is passionate about open-access, open-science, and reproducibility and is currently an ambassador ASAPBio, Protocols.io, and eLife.

Edward Emmott is a postdoctoral research associate in the Slavov lab at Northeastern University in Boston, Massachusetts. Ed earned his bachelor’s degree in Medical Microbiology & Virology from the University of Warwick and his doctorate in Virology from the University of Leeds. His current research interests revolve around ribosome specialization and the development of methods for single cell mass spectrometry. Prior to his current position, Ed’s work focused on virus–host interactions of human and animal pathogens, working at the University of Cambridge and Imperial College London. Ed is an eLife and ASAPbio ambassador, advocating preprints.

Benjamin Schwessinger is a Future Fellow and independent group leader at the Australian National University. Benjamin’s team works on plant–fungi interactions on multiple molecular and temporal scales. The team is currently focusing on the genome evolution of dikaryotic rust fungi in agricultural and natural ecosystems. Benjamin is a long-time advocate for open science and has been a member of eLife’s early career advisory group with a focus on “Reproducibility for Everyone” events.

Zach Hensel is the group leader of the Single Molecule Microbiology laboratory at the Instituto de Tecnologia Química e Biológica António Xavier in Portugal. Zach studies gene regulation and other problems at the single-molecule level in living microbial cells using fluorescence microscopy. He earned his B.Sc. in Physics at the University of Illinois at Urban-Champaign and Ph.D. in Biophysics from Johns Hopkins University followed by postdoctoral work at the Okinawa Institute of Science and Technology. Zach’s lab is currently focusing on new and improved methods for RNA imaging. He is an eLife and ASAPBio ambassador promoting preprint and preprint reviews.

## Values of preprints for ECRs

### Preprints accelerate science communication that facilitates ECR career progression

In the current scientific publishing system, journals are the gatekeepers of knowledge, defining when and where manuscripts get published and who can obtain access. Publication of manuscripts in journals can take an average of five months, with delays of over a year being common [10]. This significant delay is caused by peer review turnaround time, editorial decision making, publisher response times, the length of production process, and resubmission cycles of rejected manuscripts [2–5,10]. The timescales of ECR training stages are often short [68,69]. The protracted duration of traditional journal publishing can negatively impact ECRs seeking funding, promotion, and hiring.

Preprints empower authors to decide when their work is ready to be shared with the scientific community. Knowledge from early communication of findings informs on the state of the field and ECR decisions such as which lab to join [39]. The open-access policies of preprint servers facilitate this communication, with the added benefit of encouraging collaboration, informal discussion, and sharing and receiving data, a feature often unavailable on traditional publishing platforms [41].

A number of funding institutions, including the United States National Institutes of Health, United Kingdom Medical Research Council, and the European Molecular Biology Organization, take preprints into consideration in job [11,13,15,50] and funding applications [12,14,16,78], allowing researchers’ merit to be judged on the quality of their work rather than where it is published (as stated in the San Francisco Declaration on Research Assessment) [17].

### Preprints increase ECR visibility and facilitate networking

Networking is vital for ECR recognition and can increase the potential impact of ECR publications. It can also be a valuable way of receiving career development support, through peer support from ECRs or connections with mentors. Access to networking is often inhibited by the realities of ECR life today, i.e., having the time, travel authorization, and funds to travel to events to learn of cutting-edge research and to present their own work. Posting a preprint leads to a significant increase in both scientometric data (such as Altmetric attention scores, which capture mentions on social media, online, and in the news) and citations of the published paper when authors had posted the work first as a preprint [49]. Much discussion of preprints happens on social media, giving ECRs a new avenue to build their professional network.

### Preprints can help ECRs accelerate training time and optimize research design and quality

The earlier we know about research performed by peers, the earlier we can incorporate this information into our own research. Early access to knowledge and data can save months to years of ECR research and training time, reduce costs, and encourage risk taking. This means working in a more informed and efficient way, with a lower likelihood that our work is redundant with something being prepared for publication.

Preprints make cutting edge results, reagents, and methods available to ECRs [18] on short-term fellowships or starting new laboratories that previously would have only been available to close colleagues prior to publication following peer review [68]. For example, in the biophysics and fluorescence microscopy fields, preprinted methods were used well in advance of the peer-reviewed publication in sample labeling [19–20], instrument design [21–22], and image analysis [23].

### Preprints allow ECRs with limited funds to publish their findings with open access

The cost of publishing articles in journals is often multiple thousands of dollars, which can be prohibitive for ECRs and researchers from low-income economies with limited funds in which waivers are not available. Per-paper processing costs of preprints are low because they bear few editorial or administrative burdens associated with peer review; it is typically possible to cover costs of running a preprint server without article processing charges. Preprinting is a low-cost open-access mean of disseminating results, so that outputs are available to any researcher in the world, irrespective of whether countries and institution can afford journal publication or subscription fees [71].

### Preprints in public health and medical research can boost ECR research

Preprinting is increasing in many areas of the life sciences, but uptake in medical fields has been slower. Timely circulation of results has accelerated public-health research during infectious disease outbreaks by allowing quick identification of mechanisms of disease transmission [24–25]. Restrictions on data sharing (with appropriate considerations for patient privacy and other ethical concerns) or postponing release of results until after journal peer review have impeded research progress [24–26]. Funders such as the Wellcome Trust and Bill and Melinda Gates Foundation have recognized this and now require researchers to preprint work with urgent public health implications [16,27] and subsidize publication using the postpublication peer review platform from F1000 Research.

Expedited sharing of results in physiology and epidemiology as preprints can dramatically accelerate ECR research in interdisciplinary fields [24,28,31] as it has done in physical sciences [29,30]. In systems biology and systems medicine, preprints and open-access data can provide biochemical and physiological parameters that are key to development of complex multiscale computational models of human health and disease [31]. In the absence of open, diverse, and timely availability of research results, efficient use of such models that link molecular networks to cells, organs, and organ systems has been slow and challenging [32]. Accelerated release of biological results and methods for data integration will promptly inform evaluation of higher-resolution predictive computational models of human pathologies, boosting ECR research concerning personalized diagnostics and therapies [31,32].

Similarly, medical research will benefit from open innovation via open dissemination of research results as manuscripts and open databases. Clinical trials are multimillion-dollar, years-long efforts with critically important and time-sensitive research outputs. Yet more than 70% of clinical trials deposited to the US National Library of Medicine have no associated results article [37,71,72]. Preprints can be coupled to clinical trials databases such as http://clinicaltrials.gov to inform researchers in advance of journal publication, accelerating communication among basic and translational scientists, clinicians, and physicians. Archiving preprints that describe methods and parameters used in clinical trials will inform the design of other trials [33]. A data trial project aimed to make this happen [35], leading to initiation of a new preprint server, is MedRxiv [37]. This is a significant step in increasing transparency and building a sustainable culture of curating, archiving, and efficiently sharing results via preprints in public health and medical research [33,34].

### Preprints can accelerate the peer-review process to make ECRs more efficient

A typical life sciences manuscript receives feedback from two or more peer reviewers before publication. In many cases, authors ask for feedback from their lab and colleagues at their university, but there is no wider round of commenting until after publication. With a preprint, other researchers can discover the work sooner, potentially pointing out critical flaws or errors, suggesting new studies or data that strengthen the manuscript [11,33]. Public commenting on articles posted to preprint servers is uncommon (estimated at approximately 10% on bioRxiv) [6,80], although more frequent than commenting on journal articles. Feedback can also occur through email and social media platforms such as Twitter. Preprints can accelerate the peer-review process because (1) researchers can begin to respond to preprint comments before journal-solicited reviews are received, (2) researchers can submit higher-quality articles to journals after getting feedback from preprint readers, and (3) with the exception of a few journals [1], the journal peer review process remains largely opaque and confidential. If open preprint peer review were to become common practice, rereviewing of the same article could be avoided. We argue that the functions of journals to curate and evaluate research will be strengthened by effective utilization of preprints and open preprint peer reviews [40,42].

### Preprint commenting can help ECRs develop their reviewer skills

Only 20% of scientists perform 69% to 94% of the all journal-solicited peer reviews culminating to 63.4 million review hours a year, 15 million of which are spent rereviewing rejected papers [3,44]. These hours are spent at the expense of mentorship, research, and teaching every year. The increasingly interdisciplinary nature of biomedical research poses challenges for conventional peer review because manuscripts require a wider range of expert reviewers. Furthermore, the opinions of a handful of reviewers do not necessarily represent the diversity of perspectives in the scientific community [3,38,45]. Peer review does not guarantee reproducibility either, with most retractions in biomedical journals being prompted by the readership performing postpublication review [2,47]. Commenting on preprints by ECRs is an opportunity to sharpen their reviewing skills and to give them a voice in academic publishing that can expand and diversify the pool of peer reviewers. Platforms such as PREreview, Peeriodicals, Peer Community In, Prelights, Pubpeer, Academic Karma [70], and biOverlay have arisen to facilitate voluntary preprint-focused blogging and peer review [74]. Preprint advocacy platforms, principal investigators, and funding agencies can support preprint servers and implement methods to incentivize researchers to review and comment on preprints [41,42,46,77].

We strongly encourage ECRs to adopt the practice of reviewing preprints and publishing their reports. A frequent concern raised about preprint review is that it increases strain on an already overstressed peer-review system. However, preprint peer review can increase efficiency in the publication process: (1) editors can identify possible peer reviewers from those who comment on preprints (including those outside the traditional pool of reviewers); (2) preprint peer reviews can be forwarded to journals along with submissions; and (3) journals can solicit submissions from authors of preprints with reviews and/or comments demonstrating that the work is rigorous.

Looking at our respective interdisciplinary fields in computational modeling and systems biology, biophysics, genomics, biochemistry, plant sciences, mycology, and virology, we see strong, dynamic research cultures in which preprints and journal articles complement each other. Therefore, in the future, we envision an open preprint peer-review ecosystem that benefits ECRs and the scientific community as a whole by complementing journal-solicited peer review to strengthen the peer-review system and make it more efficient, accelerating the publication process and increasing constructive feedback.

### Preprints helps ECRs perform corrections via revisions

In a climate in which many journals are reluctant to update manuscripts except in the case of retraction or corrections, it can cost authors thousands of dollars to publish corrections [2]. A large number of peer-reviewed papers are retracted annually [51] that in some cases could be corrected instead to address errors in the original publication. Most preprint servers, including bioRxiv, give ECRs the platform to rapidly publish manuscript addenda, such as corrections and new data sets, that supplement manuscripts as new preprints while keeping the original manuscript as vital history of a research project [80]. Preprint servers also permit attachment of supporting materials and resources that exceed limitations imposed by some journals. Versioning of manuscripts by authors to narrate research progress [43,52] can be a tool to increase the likelihood that articles are accurate and reproducible. For instance, bioRxiv allows the posting of “Confirmatory Results” or “Contradictory Results” types of articles that encourage the availability of replicate studies to confirm previously published work.

### Publishing all research findings and conditions in preprints can benefit ECRs

Negative results, which are excluded data, unreported measures, and conditions [53,54] are an important output of research [55,79], and publication of negative results can be a time-saving source of knowledge for ECRs. Despite the cost and critical implications of biomedical research and clinical trials, most are not subjected to independent reanalysis, which would require deposition of all data, including negative results. Sharing of these results is uncommon, and these findings are hard to publish because their inclusion in manuscripts is often discouraged by journal editors and peer reviewers [56]. Novelty is often a prime criterion for being published in a journal, so it can be difficult for authors to publish null findings or replication studies. Writing separate manuscripts dedicated to negative results takes up substantial researcher time. A number of journals have emerged that are solely dedicated to publishing negative results [54], but the number of submissions to these journals have remained low, resulting in one such journal shutting down [53]. Preprints offer a platform to publishing negative results.

### Perceived concerns by ECR on preprinting

A number of researchers, including ECRs, have voiced perceived concerns about preprinting. We review these concerns, noting that they need to be balanced against the benefits of preprints.

**“Preprinting leads to scooping.”** Preprints can be seen as a timestamp because they are posted publicly with a digital object identifier (DOI), becoming a permanent part of the scholarly record that should be referenced in journal publications [62,75]. We encourage all journals to explicitly state in instructions to authors that preprints are citable. There is the potential that another lab with more resources could accelerate publication for competing work or repeat an interesting experiment from a preprint and publish it before the preprint’s authors do so [57]. Yet, several journals now provide scoop protection for preprints and acknowledge the importance of being second [58–60]. We note that, in many fields, it takes years to conceive ideas, perform, and finalize projects for publication. Moreover, multiple independent labs reaching the same conclusion around the same time is a sign of the reproducibility and soundness of the finding and should only be supported, not penalized, by any scientist, journal, or funding agency. Furthermore, this concern of scooping predates preprints.

**“Preprinting prevents publication.”** Most life science journals now accept submissions of preprinted manuscripts [61–63,73]. Yet, some journals have not adapted preprint-friendly policies or have confusing or self-contradictory policies. For example, one publisher’s preprint policy states that “authors can share their preprint anywhere at any time,” including updating preprints after peer review, whereas the policy for one journal published by this publisher states that “we do not support posting of revisions that respond to editorial input and peer review.” Clarity from publishers and mandates from funders on preprints will reduce ECR uncertainty [62]. We recommend that journals choose one of a few easy-to-understand preprint policies following the models used for open-access publications and for open-access publication licenses. For example,

- Preprint ɑ (PPɑ): Preprint OK; updates to preprint after review OK; preprint automatically linked from journal article
- PPβ: Preprint OK; updates to preprint after review OK
- PPɣ: Preprint OK; reviewer/editor comments cannot be used to improve preprint
- PPδ: No preprinted manuscripts accepted

To increase preprinting frequency, journals can follow the lead of PLOS, ASM, and others by making it simple for authors to automatically post submitted manuscripts to a preprint server (bioRxiv or equivalent). PLOS journals further link back to the preprint from the PLOS article, providing useful information about manuscript history [66–67]. The reverse of this policy is accepting direct submissions from preprint servers, currently implemented by many journals (PLOS journals, eLife, and others [66,67,73]). The international cancer genome consortia and the 4D nucleome project require submission to bioRxiv prior to journal submission. The rapid adoption of open-access policies shows that funders are influential in shaping scientific publishing [16,64]. Funders such as the Chan Zuckererg Initiative, the Bill and Melinda Gates Foundation, and the Wellcome Trust are leading the way in mandating preprints of funded work [16,64,65]; we hope that public funding agencies will follow their lead.

**“Preprints have low visibility.”** ECRs need to receive recognition for their work, and a common impression is that preprints are transiently recognized. This may be particularly true for publications reporting negative results that are under-appreciated in journals. In response to this, we argue that the online conversation around preprints is already more robust than that around journal articles [49], and that even if it were the case, any publishing option will benefit ECRs who need to prove productivity over a short period of time [68]. We note that bioRxiv already receives over 4 million visits per month and that the increased rate of preprinting and the development of search engines to make it easier to identify relevant preprints, such as prepubmed.org, mean that visibility will likely increase.

## Conclusions

Preprints are already benefiting ECRs and life scientists at large, but we argue that they are underutilized and can be used in new ways to aid ECR development and increase the efficiency of scientific research. Preprints empower trainees and amplify their voices, improving their graduate, postdoctoral, and early faculty experience by allowing others to learn about their work and helping ECRs form a professional network that can provide feedback, support, and opportunities. We urge trainees to embrace preprints and hope that principal investigators and senior researchers encourage posting and reviewing of articles on preprint servers. The open-access feature means that preprints can raise public awareness of health and medical research in all countries, especially in developing nations where researchers struggle to gain institutional funds to publish, read, and subscribe to scientific journals. Preprints also support an exciting and stimulating research culture. A strong preprinting culture can significantly reduce the negative impacts of the current publishing system on ECR work and life. It is time for our research communities and, more broadly, the biomedical research enterprise to embrace preprints to their full potential.

## Abbreviations

DOI: digital object identifier
ECR: early career researcher
